# The Bugaboo of Small-Pox

**Published:** 1897-03

**Authors:** M. R. Leverson

**Affiliations:** Fort Hamilton, Long Island, N. Y.


### THE BUGABOO OF SMALL-POX.

M. R. Leverson, M. D., Fort Hamilton, Long Island, N. Y.

"If no mischief be done by physician or nurse, small-pox is the most light and safe of all diseases." (Sydenham's letter to Boyle, April 2d, 1688.) Sydenham is known as "The Father of modern medicine," "The great physician," "The English Hippocrates."

There is abundant evidence to prove that of all the *filth* diseases small-pox was the lightest till the doctors made it a great cause of death, first by inoculation and then by vaccination.

In 1792 there was a tremendous epidemic of small-pox in Boston, Mass. In a population of less than 20,000 there were no less than 8,346 cases of small-pox within a period of one year, but of these 8,346 cases, 8,114 were given by inoculation! (Report No. 153 presented to the House of Representatives of Massachusetts, March 13th, 1861, section on small-pox, p. 10.)

Small-pox properly treated is neither dangerous nor *infectious;* none but infants or persons of enfeebled vitality should die of it.

Slight cases can be cured in two or three days; severe cases in from five to eight days; both classes can be cured without the smallest danger of infection. No one need hesitate to nurse those sick with it, if attended by a physician who knows how to cure it, which unfortunately few of them do.

Scare is the chief element of danger, and is wholly groundless.

Read the proceedings of the Medico-Legal Society of November, 18th, 1896, to be had of the Anti-Vaccination Society of America, Fort Hamilton, Long Island, N. Y., M. R. Leverson, M. D., Secretary.

[The above statements of Dr. Leverson are, to say the least, very surprising, not alone to the editor of this journal, but to the readers as well. We would be glad to have Dr. Leverson inform us what means he would use to render small-pox so harmless.—Ed.]

